# Cybervictimization, Self-Concept, Aggressiveness, and School Anxiety in School Children: A Structural Equations Analysis

**DOI:** 10.3390/ijerph17197000

**Published:** 2020-09-24

**Authors:** Raquel Escortell, Beatriz Delgado, María C. Martínez-Monteagudo

**Affiliations:** 1 Faculty of Education, International University of La Rioja, 26006 Logroño, Spain; raquel.escortell@unir.net; 2 Department of Developmental Psychology and Didactic, University of Alicante, 03690 Alicante, Spain; maricarmen.martinez@ua.es

**Keywords:** cybervictimization, self-concept, aggressiveness, school anxiety, Primary Education

## Abstract

The rapid increase in cases of cybervictimization amongst children has led researchers to examine the psychoemotional factors related to cyberbullying behavior, in an attempt to prevent and minimize its impact. The objective of this study was to establish and contrast the fit of an explanatory model on cybervictimization based on its relationship with self-concept, aggressiveness, and school anxiety using a structural equations analysis. A total of 542 Spanish students aged 10–12 (*M age* = 10.97; *SD* = 0.74) completed a battery of questionnaires. An adjusted structural equations model was obtained (χ^2^ = 512.23; *df* = 99; *p* < 0.001; CFI = 0.928; NFI = 0.91; IFI = 0.928; RMSEA = 0.078). A direct and negative relationship was obtained between cybervictimization and self-concept and between cybervictimization and school anxiety. In addition, a direct and positive relationship was found between aggressiveness and self-concept and between aggressiveness and school anxiety. Indirect relationships were not found between the variables. The study’s findings demonstrate that the variables of self-concept and school anxiety are directly related to cybervictimization and that the improved psychoemotional adjustment of the youngest students may help to prevent the risk of being victimized over the Internet.

## 1. Introduction

The widespread use of the Internet by children and adolescents has revolutionized access to information, leisure and entertainment, communication, etc. [1]. However, advances in the Information and Communication Technologies (ICTs), as well as their popularity amongst the youngest of users has created new opportunities for bullying and an increase in electronic victimization behaviors [2,3,4].

Cyberbullying has become one of the most frequent psychosocial problems of childhood and adolescence [5] having prevalences ranging from 6% to 72% [6,7,8]. This high variability may be explained by the distinct forms of construct conceptualization, and the distinct methodologies used for variable measurement. Therefore, although considerable debate exists with regard to its definition, measurement, and differentiation from traditional bullying [9], most studies coincide that cyberbullying may be defined as a behavior involving the use of mobile phones or the Internet to threaten or intimidate another [2,10]. Subjects involved in cyberbullying take on different roles. The most parsimonious grouping includes the profiles of victim, bully, observer, and victimized bully [11].

Cyberbullying has devastating effects on all of these roles, but especially on the victims [12]. Victim distress is often the result of the bully’s anonymity and the rapid dissemination of the harassment and the victim’s loss of control [3]. Specifically, cybervictimization has been associated with emotional distress [13], social anxiety [4,14], depression [15], a poorer quality of life with regard to health [16], and suicidal thinking [17]. Additionally, past evidence has suggested that traumatic experiences such as victimization between peers may continue to have an impact in adulthood [18,19], including anxiety disorders and depression [20]. This evidence has led to diverse studies attempting to analyze the psychoemotional and social factors explaining cybervictimization in childhood and adolescence.

### 1.1. Self-Concept and Cybervictimization

Self-concept is defined as a set of hierarchically organized perceptions held by an individual, which are based on their experience and relationships with the environment and that are influenced by support and assessments of other significant individuals and by attributions of the individual’s own behavior [21]. Self-concept plays a major role in youth’s healthy development and emotional well-being [22], in addition to being affected by and affecting social behavior and interpersonal relationships during childhood and adolescence [23]. Therefore, it has been directly and negatively related to the acceptance of aggressive behavior [24] and school anxiety during adolescence [25].

As for its relationship with cybervictimization, past studies have associated a poor self-concept with being cyberbullied by peers in preadolescent and adolescent samples [26,27]. Along these lines, Cole et al. [28] found that, in a sample of 827 US students aged 8–13 who participated in a longitudinal study, cybervictimization significantly predicted an increase in negative self-perceptions, as well as cognitive reactions related to depression. Similarly, a recent study using a sample of 548 students aged 10–12 revealed that low levels of social self-concept (relationship with parents and relationship with peers), academic self-concept (self-concept in Language), and general self-concept predict being a victim of cyberbullying [29].

Similarly, studies using structural equations models have offered similar results. Jenkins and Demeray [30] found that, in a sample of 1413 adolescents in the US, a negative and significant relationship existed between electronic victimization and self-concept. Along this same line, Blakely-McClure and Ostrov [31], using a sample of 1063 US students aged 10–15, found that relational victimization was associated with self-concept in three domains (academic, sporting, and physical appearance). In addition, these effects appear to have an extensive impact, as concluded by Norrington [18], whose study with 1413 adolescents found that self-concept partially mediated the relationship between victimization and psychological problems during adulthood.

### 1.2. Aggressiveness and Cybervictimization

Bushman and Anderson [32] define aggressiveness as a behavior having the intent of causing harm to another, in which the aggressor previously believes that his/her behavior will harm the victim and that this individual will attempt to avoid said behavior.

In general, aggressive behavior has been negatively related to personal adjustment and self-concept during adolescence [33,34]. Along this same line, and based on a structural equations analysis, Castro-Sánchez, Zurita-Ortega, Ruiz-Rico, and Chacón-Cuberos [35] found that manifest aggressiveness is negatively related to self-concept, while relational aggressiveness is related to affective empathy.

As for its relationship with cyberbullying, while past studies have associated high levels of aggressiveness with the role of the bully [36,37] and the victimized bully [38], they have also been related with that of the victim [39,40]. Some studies have suggested that cybervictimization is positively associated with higher rates of anger. Lonigro et al. [41], examining 715 Italian students aged 11–19, found that aggressiveness in the form of anger is directly related to cybervictimization. Similarly, Wei and Williams [42], in a study with 219 secondary students from Taiwan, revealed that relational aggression is associated with cybervictimization. Along the same line, and based on structural equations analysis, Ak, Özdemir, and Kuzucu [43], using a sample of 687 Turkish university students, found that cybervictimization is positively related to anger and indirectly related to cyberaggression through anger.

### 1.3. School Anxiety and Cybervictimization

School anxiety consists of a set of symptoms grouped together as cognitive, psychophysiological, and motor-based responses of a subject in academic situations that are perceived as threatening [44]. Therefore, students with school anxiety have serious difficulties in attending or remaining in school on a regular basis given an excess or irrational fear associated with diverse academic situations [45]. Studies conducted over recent years have suggested that school anxiety is a variable that impacts the psychosocial and scholastic development of minors, relating it with poor scholastic motivation [46], poor academic performance [45], and higher levels of state anxiety, trait anxiety, and depression [47].

As for its relationship with cyberbullying, the limited empirical evidence existing supports a positive relationship between anxiety and cybervictimization. Delgado, Escortell, Martínez-Monteagudo, and Aparisi [48], in a study with 548 students aged 10–13, found that high scores on anxiety regarding social assessment explained being cybervictims. In addition, the students who were victims of cyberbullying displayed more symptoms of anxiety in distinct academic situations than students who were victimized bullies and those who were not involved in the cyberbullying cases [48]. González-Cabrera, Calvete, León-Mejía, Pérez-Sancho, and Peinado [49], upon analyzing the levels of cortisol release, found that anxiety and perceived stress predicted cybervictimization. On the other hand, Halabi, Ghandour, Dib, Zeinoun, and Maalouf [50], examining 510 Lebanese students aged 11–17, concluded that one of the factors shown to be the greatest predictor of cyberbullying was the presence of an anxiety disorder. These findings were also supported by Sjurso, Fandrem, and Roland [51] who, using a structural equations analysis on a sample of 1583 Norwegian adolescents, found an association between cybervictimization and general symptoms of anxiety, more so than in traditional bullying. Finally, Resset and Gámez-Guadix [52], using a sample of 898 adolescents, found that both cybervictimization and cyberaggression are related to symptoms of anxiety.

### 1.4. This Study

As seen in the reviewed studies, cyberbullying causes major harm to its victims. Most of the studies use adolescent samples, despite the fact that the scientific literature has shown that cyberbullying also takes place during late childhood [53,54,55]. Therefore, the assessment of cybervictimization needs to also be carried out at early ages, in order to attempt to explain the psychoemotional profile of the cyberbullying victims, and thereby prevent potential bullying situations. Additionally, although self-concept, aggressiveness, and school anxiety are very important variables in psychoemotional adjustment of children, currently, no studies have analyzed whether or not they collectively explain cybervictimization or whether feedback exists between them.

Therefore, and given the many unanswered questions remaining in this area with regard to primary education students, this work proposes to define and contrast a structural equations model to analyze the relationship between self-concept, aggressiveness, school anxiety, and cybervictimization in students aged 10–12.

Based on previously reviewed studies, it is expected that self-concept will be negatively associated with cybervictimization (Hypothesis 1). Moreover, it is anticipated that aggressiveness will be positively related to cybervictimization (Hypothesis 2), and that school anxiety will also be positively associated with cybervictimization (Hypothesis 3). Finally, self-concept is expected to negatively relate to aggressiveness (Hypothesis 4) and school anxiety (Hypothesis 5), and aggressiveness is expected to positively relate to school anxiety (Hypothesis 6).

## 2. Materials and Methods

### 2.1. Participants

After probabilistic sampling of the public and private schools of the Alicante (Spain) province, a total of six primary education schools participated in the study. The sample consisted of a total of 542 students aged 10–12 (*M* = 10.97; *SD* = 0.74), of which, 50.2% were male and 49.8% were female. The distribution according to grade was 273 (52.8%) 5th graders and 269 (47.2%) 6th graders. A χ^2^ test was performed to analyze the homogeneity of the sample, considering sex and grade, finding no statistically significant differences between the four sex x grade groups (χ^2^ = 2.53; *p* > 0.05).

### 2.2. Instruments

Cybervictimization. The Screening of Harassment among Peers (SHP) [56] questionnaire was used to measure electronic bullying. This is a self-reporting tool that permits the assessment of face-to-face behavior (Bullying subscale) as well as electronic bullying behavior (Cyberbullying subscale), both for victims, bullies, and bystanders. In this study, only responses related to the role of cybervictim were considered. This subscale assesses 15 harassment behaviors (e.g., “Have you been assaulted to be recorded and posted on the Internet?”) via electronic means (sending offensive or insulting messages, making offensive calls, spreading photographs or videos on YouTube, making anonymous calls to scare, blackmail, or threaten, etc.), which are responded to using a four-point Likert-like scale, ranging from 0 to 3 (0 = *never*; 3 = *always*). The psychometric studies performed by the original authors confirm an appropriate internal consistency (*α* = 0.91) and a factorial structure that explains 40.15% of the construct variance [56]. Similarly, past publications have supported the reliability and validity of the instrument [57,58]. The internal consistency rate of the Cybervictimization subscale used in this study was found to be adequate (α = 0.94).

School anxiety. The School Anxiety Inventory for Primary Education (SAI) [59] is an instrument that measures school anxiety responses of students aged 8–12, considering four factors (Anxiety related to assessment and academic failure; Anxiety related to victimization; Anxiety related to social assessment; Anxiety related to scholastic punishment). The questionnaire consists of 57 items referring to declarations of anxiety in 19 academic situations (e.g., “If the teacher asks for my homework and I haven’t done it, I feel guilty”), and are responded to using a Likert-like scale ranging from 0 to 4 points (0 = *never*; 4 = *always*). Psychometric characteristics were analyzed in the original study with a sample consisting of 309 students aged between 8 and 12 [59]. The coefficients of internal consistency of the factors were satisfactory: α = 0.83 (Anxiety related to victimization); α = 0.88 (Anxiety related to assessment and academic failure); α = 0.84 (Anxiety related to social assessment); α = 0.85 (Anxiety related to scholastic punishment). In this study, the following rates of internal consistency were found for the IAEP subscales: Anxiety related to victimization α = 0.88; Anxiety related to assessment and academic failure α = 0.98; Anxiety related to social assessment α = 0.85; Anxiety related to scholastic punishment α = 0.90.

Self-concept. The Self-Description Questionnaire (SDQ-I) [60] is an instrument designed to measure the multidimensional self-concept of children aged 7–12. It consists of 76 items and uses a Likert-type response (0 = *no*; 4 = *yes*), distributed over seven subscales: Physical ability (e.g., “I am a fast runner”), Physical appearance (e.g., “I like my physical appearance”), Relationship with peers (e.g., “I make friends easily”), Relationship with parents (e.g., “My parents understand me”), Self-concept in language (e.g., “Language class interests me”), Self-concept in mathematics (e.g., “I am good at math”), and General self-concept (e.g., “In general, I like how I am”). Marsh [61] developed the instrument based on the multidimensional and hierarchical self-concept model of Shavelson, Hubner, and Stanton [21]. The Spanish adaptation was made by González, Torres, Tourón, and Gaviria [62] using a sample of 674 5th graders in primary education, obtaining adequate reliability indices (α = 0.90). In this study, the internal consistency coefficients (Cronbach’s alpha) obtained ranged from between 0.82 (Mathematics self-concept) and 0.71 (General self-concept).

Aggressiveness. The Aggression Questionnaire (AQ) [63] consists of 29 items using Likert-like responses for four factors: Physical aggression (e.g., “I have physically threatened others”), Verbal aggression (e.g., “I fight with others a lot”), Anger (e.g., “When I am frustrated, I tend to show my irritation”), and Hostility (e.g., “Sometimes I feel that people criticize me behind my back”). The Spanish adaptation was made by Andreu, Peña, and Grana [64] using a sample of 1382 students. The results revealed a factorial structure consisting of four factors that explained 46.3% of the total variance, with adequate reliability indices: α = 0.86 (Physical aggression); α = 0.77 (Anger); α = 0.68 (Verbal aggression); α = 0.72 (Hostility). The reliability coefficients for the AQ scores in this study were acceptable: Physical aggression (α = 0.77); Verbal aggression (α = 0.74); Anger (α = 0.69); Hostility (α = 0.75).

### 2.3. Procedure

After an interview with directors and school counsellors from the selected schools, intended to inform them of the study objectives and request the relevant permission, an authorization was requested from the parents of the minors in order to request their signed informed consent. Tests were carried out collectively and voluntarily, in two class sessions, ensuring participant anonymity and confidentiality. The study researchers, along with the students’ teachers, were present during questionnaire completion in order to clarify any potential doubts and to ensure the correct development in its implementation. All standards on research conducted on humans were respected, in accordance with the ethical principles of the Helsinki Declaration, and were approved by the University’s Ethics committee (UA-2018-02-21).

### 2.4. Statistical Analysis

IBM SPSS (version 22.0, IBM Corporation, Armonk, NY, USA) statistical software was used to perform the basic descriptive analyses and Pearson correlation coefficients. For analysis of the potential relationships existing between study variables, a structural model was designed to establish the relationships between the constructs intervening in the study. These data were calculated using the IBM AMOS 23 program to obtain the covariance matrix of the examined variables. The program is useful for these analyses, since the main study constructs are made up of various observed variables [65]. After verifying that the distribution of the scores was normal [66], the Maximum Likelihood method was selected.

**Figure 1:**
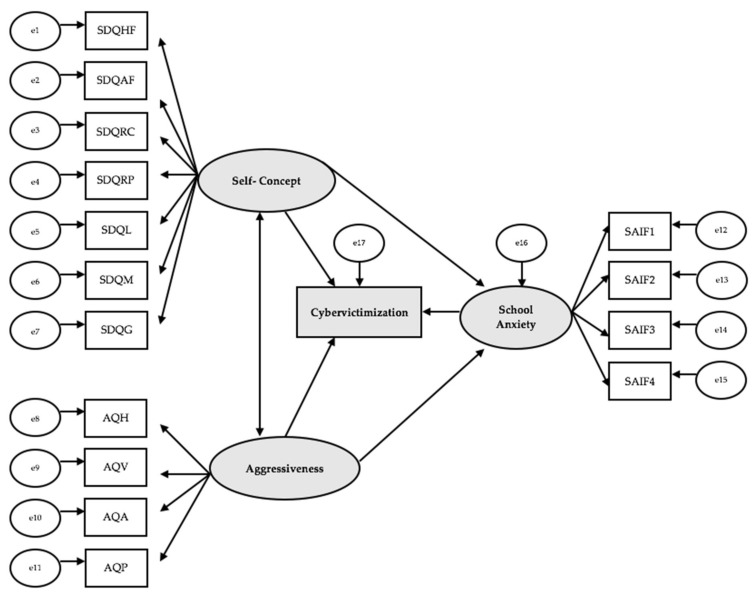
Model theories. Note: SAIF1: Anxiety related to assessment and academic failure; SAIF2: Anxiety related to victimization; SAIF3: Anxiety related to social assessment; SAIF4: Anxiety related to scholastic punishment; AQH: Hostility; AQV: Verbal aggression; AQA: Anger; AQP: Physical aggression; SDQHF: Physical ability; SDQAF: Physical appearance; SDQRC: Relationship with peers; SDQRP: Relationship with parents; SDQL: Self-concept in language; SDQM: Self-concept in mathematics; SDQG: General self-concept.

The complete structural equations model consists of 16 observed variables and three latent variables to measure the indicators (see Figure 1). In this model, the causal explanations of the latent variables are formulated based on the relationships observed between indicators, taking into account the reliability of the measurements. Measurement errors are also included in the model, permitting their direct control. Unidirectional arrows are lines of influence between the lateral and observable indicators and are interpreted as multivariate regression coefficients. Bidirectional arrows reveal the relationship between latent variables, which also represent regression coefficients.

Self-concept acts as an exogenous variable and influences seven indicators: Physical ability (SDQHF), Physical appearance (SDQAF), Relationship with peers (SDQRC), Relationship with parents (SDQRP), Self-concept in language (SDQL), Self-concept in mathematics (SDQM), and General self-concept (SDQG). Aggressiveness also acts as an exogenous variable, influencing four indicators: Hostility (AQH), Verbal aggression (AQV), Anger (AQA), and Physical aggression (AQP). School Anxiety as an exogenous variable influences four indicators: Anxiety related to assessment and academic failure (SAIF1), Anxiety related to victimization (SAIF2), Anxiety related to social assessment (SAIF3), and Anxiety related to scholastic punishment (SAIF4). On the other hand, School Anxiety also acts as an endogenous variable, receiving the effect of Self-concept and Aggressiveness. In addition, Cybervictimization receives the influence of Self-concept, Aggressiveness, and School Anxiety, thereby acting as an endogenous variable.

Bootstrap analysis was also used to estimate the 95% confidence intervals with bias correction for the direct and indirect effects of School Anxiety on the association between Self-concept and Cybervictimization, as well as between Aggression and Cybervictimization.

To verify the compatibility of the proposed model and the empirical data obtained, verification of the model’s fit was performed. Goodness of fit was calculated using diverse indices proposed by Marsh [67]. In the case of the Chi-squared test, the nonsignificant values associated with *p* indicate a good model fit. However, normalized adjustment indices were also calculated, which are not sensitive to sample size. Therefore, the Comparative Fit Index (CFI) is considered acceptable for values exceeding 0.90 and excellent for values exceeding 0.95. The Normalized Fit Index (NFI) should be greater than 0.90. The value of the Incremental Fit Index (IFI) is considered acceptable for values exceeding 0.90 and excellent for values higher than 0.95. Finally, the Root Mean Square Error of Approximation (RMSEA) is considered acceptable if lower than 0.08 and excellent if lower than 0.05.

## 3. Results

In total, 31.7% of the students affirm that they have been cyberbullied on at least one occasion during the past year. The most frequent cybervictimization behaviors were as follows: receiving offensive or insulting messages via mobile phone or the Internet (16.6%), having their blog or email passwords stolen (9.3%), being defamed over the Internet (8.5%) and receiving anonymous calls to frighten them (8.3%) or receiving messages containing threats or blackmail over the mobile phone (6.1%). Table 1 shows the correlations between subscales of the Cybervictimization, School Anxiety, Aggressiveness, and Self-Concept variables and the descriptive statistics.

The proposed structural equations model revealed a good fit in all of the assessment indices. The Chi-squared test revealed a significant value of *p* (χ^2^ = 512.23; *df* = 99; *p* < 0.001). This index, however, cannot be interpreted in this standardized manner, and there is also the problem of its sensitivity to sample size [67]. Therefore, other normalized adjustment indices were used, which are less sensitive to sample size. The Comparative Fit Index (CFI) obtained an acceptable value of 0.928. The Normalized Fit Index (NFI) resulted in a value of 0.913, and the Incremental Fit Index (IFI) had a value of 0.928, both of which are acceptable. The Root Mean Square Error of Approximation (RMSEA) also had an acceptable value of 0.078. The variables included in the structural equations model explain 67% of the Cybervictimization construct.

**Figure 2:**
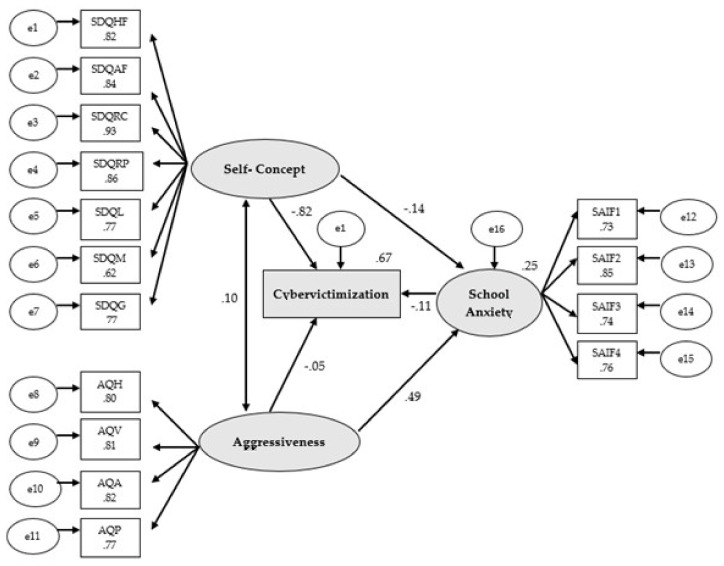
Structural equations model. Note: SAIF1: Anxiety related to assessment and academic failure; SAIF2: Anxiety related to victimization; SAIF3: Anxiety related to social assessment; SAIF4: Anxiety related to scholastic punishment; AQH: Hostility; Hostility; AQV: Verbal aggression; AQA: Anger; AQP: Physical aggression; SDQHF: Physical ability; SDQAF: Physical appearance; SDQRC: Relationship with peers; SDQRP: Relationship with parents; SDQG: General self-concept.

In Figure 2 and Table 2, an estimate of the model parameters is presented. Factorial loads of the indicators corresponding to the latent variables (Self-concept, Aggressiveness, and School Anxiety) and observed variables (Cybervictimization) are mainly significant. Statistically significant relationships are observed (*p* < 0.001), which are positive and direct, amongst all of the Self-concept dimensions. In addition, there are positive and direct relationships between Aggressiveness and its dimensions (*p* < 0.001), as well as between School anxiety and the indicators making it up (*p* < 0.001), with all of the associations being positive and direct.

Upon analyzing the factorial loads of the indicators corresponding to latent variables, it may be observed that they all have statistically significant differences at a level of *p* < 0.001, with direct and positive relationships. As for Self-concept, relationship with peers (*r* = 0.93) is the indicator having the highest coefficient, followed by relationship with parents (*r* = 0.86), physical appearance (*r* = 0.84), physical ability (*r* = 0.82), Self-concept in language and general self-concept (*r* = 0.77) and, ultimately, Self-concept in mathematics (*r* = 0.62), having the lowest levels. For Aggressiveness, the greater association was found in anger (*r* = 0.77), followed by verbal aggression (*r* = 0.81), hostility (*r* = 0.80) and with a lower association, physical aggression (*r* = 0.77). Finally, the factors that best make up School Anxiety are anxiety related to academic assessment (*r* = 0.76) and anxiety related to social assessment (*r* = 0.74).

In addition, significant associations are observed between the variables (*p* < 0.001). Therefore, Self-concept is negatively and directly related to Cybervictimization (*r* = −0.82). Along the same lines, the relationship between School Anxiety and Cybervictimization is negative and direct (*r* = −0.11), with no statistically significant association being found between Aggressiveness and Cybervictimization (*p* = 0.186). Finally, a direct and negative relationship has also been observed between Self-concept and School Anxiety (*r* = −0.14) and a direct and positive relationship is seen between Aggressiveness and School Anxiety (*r* = 0.49). Statistically significant indirect effects have not been observed (*p* > 0.05) between Aggressiveness and Cybervictimization (*r* = −0.053) and between Self-Concept and Cybervictimization (*r* = 0.015) through School Anxiety. Therefore, School Anxiety does not act as a mediator in the cited relationships.

## 4. Discussion

The objective of this study is to analyze the relationship between self-concept, aggressiveness, school anxiety and cybervictimization in students aged 10–12. Based on the first hypothesis, the structural equations model confirms the existence of a direct, negative, and statistically significant relationship between self-concept and cybervictimization. That is, the low level of self-concept explains being a cybervictim, thereby confirming hypothesis 1. This evidence is congruent with the results of Jenkins and Kilpatrick [30] and Blakely-McClure and Ostrov [31], who used path analyses to indicate that self-concept and cybervictimization are negatively associated. This is consistent with studies that have also found this relationship in the specific dimensions of self-concept, such as academic self-concept [26], self-concept referring to relationships with parents and peers [29], and general self-concept [26,27,29]. Therefore, negative self-perceptions during preadolescence may be risk factors for becoming a cybervictim. Specifically, students having a low self-concept may become easy targets for cyberbullies since their profile is characterized by insecurity, fear, and personal mistrust [2]. In addition, the effort to make up for the consequences of a poor self-concept, along with the anonymity and disinhibition found in screen-based communication [3], makes these minors more likely to use virtual environments [5], increasing their probability of becoming cybervictims [2].

As for the relationship with aggressiveness, a positive association with cybervictimization is expected (hypothesis 2). However, our results did not support this hypothesis. Evidence is contradictory to that of other past studies that have found that aggressiveness, in the form of anger, is directly related to cybervictimization [41,42,43]. The dissonance of these results may be due to differences in ages of the samples since most of the prior studies used adolescent samples. Since bullying behavior has been shown to begin in primary school [53,54,55], it is likely that the high scores on aggressiveness develop with time of exposure and not during the initial contacts, which may lead to an overlapping of roles in subsequent grades and the development of a victimized bully role, whose aggressiveness indices have been found to be higher [38,68]. Similarly, it is important to consider the perspective of students with regard to aggressive behavior, given that for minors, some types of aggressiveness, such as verbal, are not considered to be cyberbullying behaviors, since they are used to facilitate communication and interaction with their peers, which would explain why all declarations of aggressiveness are not related to the cybervictim role [69].

As for school anxiety, the results suggest the existence of a negative and significant association between the variables, thereby rejecting hypothesis 3. These results are contrary to those of past studies, which have situated high anxiety as a variable that explains cybervictimization [50,51], possibly due to the fact that most past studies have focused their attention on the social anxiety construct [70] and, in this case, anxiety is confined to the academic environment. Therefore, and considering Lang’s theory [71], anxiety is understood to be a set of cognitive, psychophysiological, and behavioral symptoms triggered by internal or external stimuli. This response may vary based on the type of triggering stimuli. Therefore, social anxiety is characterized by an intense fear of social situations [72], while school anxiety is confined to the assessment, punishment, and relations focused on school performance [45]. As for cybervictimization, high school anxiety may lead students to avoid risky situations in virtual environments in order to avoid situations that cause anxiety, such as punishment, reprimands, or negative assessment by peers, parents, and teachers [48]. In any case, the school anxiety construct is multidimensional; thus, distinct anxiety-based manifestations and situations in scholastic contexts, which are related in the same direction as the cybervictimization phenomenon, should be analyzed in greater depth.

On the other hand, self-concept is shown to be positively related to aggressiveness, and therefore, hypothesis 4 was not supported. The results are contradictory to those of Esteve et al. [24], who affirmed an inverse relationship between both constructs, with low scores in self-concept generating acceptance of aggressive behaviors, in accordance with past studies [33,34,35]. In the case of students from the sample, the opposite was found, with high scores on self-concept leading to higher levels of aggression. In this case, the data suggests that preadolescents having higher levels of aggressiveness developed a greater self-concept, possibly helping to protect them from cybervictimization. These findings may be supported by the evidence from the correlational analysis, since some self-concept dimensions, but not all of them, may be positively related with manifestations of aggression. Therefore, some studies have detected that minors with a positive physical or social self-image may be related to social behaviors of dominance over their peers [73,74], suggesting an aggressive interpersonal relationship style.

Self-concept has been found to be negatively associated with school anxiety, confirming hypothesis 5. These findings are consistent with Gonzálvez et al. [25], who showed that adolescents having low self-concept scores had significantly higher scores on school anxiety. Therefore, it is found that adolescents having higher levels of school anxiety are less likely to positively perceive their social relations with their peers or parents, in addition to perceiving themselves as being less attractive or athletic, more emotionally unstable, and having lower self-esteem and academic ability. Ultimately, this profile is quite characteristic of the cyberbullying victim role, as revealed in past studies [29,30].

The positive and significant relationship between aggressiveness and school anxiety is in line with past studies, and thereby confirms hypothesis 6. Therefore, as suggested by past works, students with a moderate profile and high level of school anxiety declared to have suffered from more episodes of scholastic violence [75], as well as situations of aggression amongst peers [25,76]. In this study, aggressiveness directly and positively explained the levels of school anxiety, although this relationship was not significant as a mediating variable.

In general, the structural equations model created adjusted adequately and explained 67% of the cybervictimization phenomenon, based on two latent variables: self-concept and school anxiety. Both self-concept and school anxiety acted as protective factors against electronic bullying. In addition, it has been shown that the interaction between aggressiveness, self-concept, and school anxiety is significant, but the latter does not act as a mediating variable for aggressiveness with regard to cybervictimization.

This study has various limitations, mainly the limited number of works on cybervictimization in primary school education, which hinders the comparison of its results with those of past studies. In addition, given the study’s cross-sectional design, it is impossible to establish causal relationships. Therefore, new research lines are available for longitudinal studies that may offer information over time. Finally, there is a lack of consensus in terms of defining the cyberbullying concept, making it necessary to update the same, given the constant advances taking place in the ICT. Finally, it is important to note that the study focuses only on the role of victim, which does not allow us to assess the possible relationship between the variables and the other figures involved in cyberbullying.

## 5. Conclusions

This study offers novel and relevant information on the study of cyberbullying, specifically in the explanation of the role of the cybervictim. On one hand, it demonstrates the existence of cybervictimization at early ages, and, on the other hand, it highlights the risk and protection factors that explain being bullied with ICT. Specifically, minors having higher levels of self-concept may be more protected from becoming online victims. Additionally, high levels of school anxiety may also explain lower participation, suggesting that some scientific conclusions referring to secondary students may not be fully applicable to younger students. In addition, aggressiveness was not found to be an explanatory variable for cybervictimization, although it did have a direct relationship with self-concept, revealing that, at these young ages, being a bully may be related to having a positive self-image which, at the same time, may decrease the risk of being cybervictimized. In addition, aggressiveness positively explained the high scores received on school anxiety. All of this reinforces the need to conduct prevention campaigns during later years of primary school education to promote self-concept, so as to ensure better psychological adjustment and personal competence, helping youth develop an adjusted scheme of themselves and thereby empower them in the face of potential cyberbullying situations. In addition, it also offers relevant information about the variable aggressiveness and school anxiety, as well as the relationship between both, with great relevance to the study of psychosocial variables in minors in order to contribute to an integral development and enhance their well-being.

Considering the most significant findings, this study opens new lines of research that include the other roles participating in the dynamics of cyberbullying, that is, bullies, bystanders, and bully-victims, in order to create differentiated profiles of each role that guide in the development of effective prevention and intervention programs in the fight against cyberbullying. In addition, it would be necessary to specify which dimensions of each variable are more related to the dynamics of each role, in order to specify the lines of action as much as possible, as well as to replicate the study with students of different educational levels.

## Figures and Tables

**Table 1:** Descriptive analyses and correlations of SAI, SDQ-I, AQ, and cybervictimization.

Variables	1	2	3	4	5	6	7	8	9	10	11	12	13	14	15	16
1. SAI: F1	1.00															
2. SAI: F2	0.51 **	1.00														
3. SAI: F3	0.51 **	0.49 **	1.00													
4. SAI: F4	0.57 **	0.46 **	0.55 **	1.00												
5. AQ: H	0.30 **	0.31 **	0.43 **	0.44 **	1.00											
6. AQ: V	0.23 **	0.17 **	0.29 **	0.23 **	0.62 **	1.00										
7. AQ: A	0.27 **	0.24 **	0.32 **	0.37 **	0.65 **	0.67 **	1.00									
8. AQ: P	0.16 **	0.08	0.24 **	0.22 **	0.62 **	0.66 **	0.62 **	1.00								
9. SDQ: HF	0.01	−0.20 **	−0.06	−0.04	0.05	0.15 **	0.11 *	0.14 **	1.00							
10. SDQ: AF	−0.02	−0.20 **	−0.12 **	−0.07	0.01	0.13 **	0.04	0.06	0.76 **	1.00						
11. SDQ: RC	0.02	−0.13 **	−0.01	−0.01	0.08	0.13 **	0.12 **	0.09 *	0.77 **	0.79 **	1.00					
12. SDQ: RP	−0.03	−0.13 **	−0.07	−0.05	−0.01	0.01 *	0.09 *	0.05	0.66 **	0.69 **	0.79 **	1.00				
13. SDQ: L	0.06	−0.07	−0.06	0.03	0.02	0.07	0.05	0.05	0.61 **	0.62 **	0.69 **	0.72 **	1.00			
14. SDQ: M	−0.05	−0.11 *	−0.17 **	−0.18 **	−0.03	−0.02	−0.01	−0.02	0.49 **	0.49 **	0.58 **	0.54 **	0.45 **	1.00		
15. SDQ: G	−0.06	−0.19 **	−0.14 **	−0.10 *	−0.08	−0.05	−0.07	−0.03	0.64 **	0.75 **	0.73 **	0.64 **	0.63 **	0.51 **	1.00	
16. Cybervictimization	−0.08	0.07	−0.02	−0.09 *	−0.09 *	−0.17 **	−0.18 **	−0.12 **	−0.66 **	−0.61 **	−0.78 **	−0.81 **	−0.68 **	−0.49 **	−0.45 **	1.00
*M*	24.64	12.91	8.68	13.70	13.58	5.67	10.38	9.84	20.54	19.53	19.42	21.74	17.74	17.02	23.73	8.97
*SD*	17.10	13.94	9.63	10.13	7.11	4.35	5.51	7.22	7.50	6.72	8.53	8.27	6.70	7.69	5.08	11.68
Min	0.00	0.00	0.00	0.00	0.00	0.00	0.00	0.00	1.00	2.00	4.00	0.00	2.00	0.00	5.00	0.00
Max	60.00	56.00	46.00	36.00	32.00	20.00	28.00	36.00	32.00	32.00	32.00	32.00	32.00	32.00	32.00	34.00

Note: * *p* < 0.01; ** *p* < 0.001. SAIF1: Anxiety related to assessment and academic failure; SAIF2: Anxiety related to victimization; SAIF3: Anxiety related to social assessment; SAIF4: Anxiety related to scholastic punishment; AQH: Hostility: Hostility; AQV: Verbal aggression; AQA: Anger; AQP: Physical aggression; SDQHF: Physical skill; SDQAF: Physical appearance; SDQRC: Relationship with peers; SDQRP: Relationship with parents; SDQL: Self-concept in language; SDQM: Self-concept in mathematics; SDQG: General self-concept.

**Table 2:** Structural model values.

Relationships between Variables			Unstandardized Regression Weights				Standardized Regression Weights
			Estimates	S.E.	C.R.	*p*	Estimates
School Anxiety	<---	Self-Concept	−0.438	0.139	−3.156	0.002	−0.143
School Anxiety	<---	Aggressiveness	1.094	0.120	9.101	***	0.491
AQP	<---	Aggressiveness	1.000				0.774
AQA	<---	Aggressiveness	0.809	0.042	19.146	***	0.820
AQV	<---	Aggressiveness	0.632	0.032	19.456	***	0.812
AQH	<---	Aggressiveness	1.014	0.054	18.714	***	0.798
SDQG	<---	Self-Concept	1.000				0.800
SDQM	<---	Self-Concept	1.164	0.076	15.304	***	0.615
SDQL	<---	Self-Concept	1.284	0.063	20.517	***	0.779
SDQRP	<---	Self-Concept	1.770	0.075	23.707	***	0.870
SDQRC	<---	Self-Concept	1.932	0.075	25.877	***	0.920
SDQAF	<---	Self-Concept	1.378	0.061	22.639	***	0.834
SDQHF	<---	Self-Concept	1.494	0.069	21.545	***	0.810
SAIF1	<---	School Anxiety	1.000				0.729
SAIF2	<---	School Anxiety	0.727	0.054	13.530	***	0.650
SAIF3	<---	School Anxiety	0.568	0.039	14.632	***	0.736
SAIF4	<---	School Anxiety	0.620	0.040	15.409	***	0.763
Cybervictimization	<---	Self-Concept	−2.448	0.139	−17.641	***	−0.853
Cybervictimization	<---	Aggressiveness	0.081	0.061	1.322	0.186	0.039
Cybervictimization	<---	School Anxiety	−0.100	0.028	−3.515	***	−0.107

Note: *** *p* < 0.001 (bilateral); SAIF1: Anxiety related to assessment and academic failure; SAIF2: Anxiety related to victimization; SAIF3: Anxiety related to social assessment; SAIF4: Anxiety related to scholastic punishment; AQH: Hostility: Hostility; AQV: Verbal aggression; AQA: Anger; AQP: Physical aggression; SDQHF: Physical ability; SDQAF: Physical appearance; SDQRC: Relationship with peers; SDQL: Self-concept in language; SDQM: Self-concept in mathematics; SDQRP: Relationship with parents; SDQG: General self-concept.
